# Medical Opinion and Movement

**Published:** 1911-01-28

**Authors:** 


### Medical Opinion and Movement.

### Hæmorrhagic Cystitis after Rectal Operations.

In a communication to the *Berliner Klinische Wochenschrift* Dr. Hadda draws attention to the frequency with which operations on the rectum are followed by blood in the urine and cystitis. Of 153 patients undergoing rectal operations ninety-four, or 61.4 per cent., had to be catheterised, and of these forty-five, or nearly 50 per cent., had urinary trouble, that is to say, four had simple cystitis, forty had hæmorrhagic cystitis, or hæmaturia without cystitis, and one had an abscess of the prostate. The hæmaturia generally appeared from the third to the sixth day after operation. In half of the cases the cystitis began with a severe hæmorrhage, with the other usual signs of inflammation of the bladder. At first the urine appeared to contain blood only, leucocytes appearing later. The hæmaturia generally ceased about the third to the fifth day, and after that there was only an ordinary catarrhal cystitis, which cleared up in the course of two or three weeks. The author attributes this complication to the anastomoses between the rectal and vesical veins, rendering easy the passage of germs from the one part to the other and facilitating thrombosis of the vesical venous plexus. He also suggests that the coprostasis which usually follows rectal operations may also cause a migration of bacilli through the intestinal walls.

### Resorcin for Chronic Conjunctivitis.

Resorcin has been employed in ocular therapeutics for a long time in the form of an ointment in cases of ciliary blepharitis, but it has been found too irritating in this form for application to the conjunctiva. According to Dr. Knapp, of Bâle, it can be used with advantage as an aqueous solution of 2 to 3 per cent. strength and instilled into the eye two or three times a day in cases of chronic conjunctivitis. The burning sensation produced passes off quickly, and the solution is efficacious when no further benefit is obtained by the application of zinc sulphate. Aqueous solutions of resorcin turn brown rather rapidly on exposure to air, especially in an alkaline medium; consequently the author advises that the resorcin be dissolved in a solution of boric acid instead of in pure water.

### Dyspepsia and Fats.

Recently the administration of fatty food has been advocated in cases of gastric trouble. Dr. Bassler reports four cases in the *New York Medical Journal*, in which the symptoms appeared to indicate this treatment with fats, but were only aggravated and intensified by it. The patients were all middle-aged, and they had been treated for some time for gastric disorders without improvement; but they rapidly got well as soon as they were put on a diet free from fat—skimmed milk, white of eggs, green vegetables, beef tea and carbohydrates, etc. They had not suffered from any previous stomach trouble. They now complained of acute pain in the epigastrium radiating through to the back. The pain was independent of food and very variable in regard to duration. There was no vomiting. Physical examination revealed no abnormal sign, even at the time of the pain. The first diagnosis was pyloric spasm, and, as already mentioned, the patients were given a fatty diet, with olive oil, which in no way relieved the condition. In one of these cases catheterism of the stomach was performed after a test meal, and about 900 c.c. of bile-stained fluid withdrawn, with a considerable amount of fat floating on the top smelling strongly rancid. As the operation was carried out early in the morning, it was evident that the fat was derived from the food ingested on the previous day. A similar result followed catheterism of the stomach in a second case. In both cases considerable relief accompanied this treatment.

### Micro-organism of Small-pox and Vaccine.

From a report on the bacteriological work carried out by Dr. M. Rabinovitch it appears probable that he has succeeded in isolating and identifying the pathogenic micro-organism of small-pox and vaccine. He was first drawn to the subject by his investigation of cases of typhus fever, from which he has been able to isolate a diplobacillus very similar to that described by Dr. Predtetchensky. In two such cases sections of the internal organs stained with silver nitrate showed the presence of certain micrococci as well as the diplobacilli in question, and he ascertained that these two patients had been vaccinated a few days before their death. Subsequently he found the same kind of micrococci in the organs and skin stained with silver nitrate of a patient who had died from small-pox. Following up these observations, he demonstrated the presence of these microbes in the internal organs and in the cutaneous pustules of twelve patients dying of small-pox. In preparations of the blood and spleen stained by Giemsa's reagent these micrococci are arranged in pairs, which are frequently joined up in chains, thus forming a diplostreptococcus. In studying the literature on the subject the author finds that similar cocci have been described by previous workers. He has found the same organism in vaccine, and has succeeded in obtaining it in pure cultures from the pustules and blood of patients suffering from small-pox and also from vaccine, and he has carried out a series of inoculations on white mice, rats, and rabbits. In certain characters the organism resembles Nöutest's diplococcus, but differs from it in others. It grows on agar or agar and glycerine, does not liquefy gelatine, and it stains with Gram. Injected subcutaneously in rabbits it causes a rise of temperature and loss of body weight, but its virulence is greater when inoculated by scarification. It is more virulent for rats, causing death in a few hours to twelve days, according to the dose and size of the animal. Inoculation by scarification produces in all the animals pustules similar to the vaccination pustules in man. Previous vaccination of the animals attenuates the reaction following inoculation with the pure cultures of the diplostreptococcus, and enables the animals to resist a considerable dose and survive.

### A New Industrial Disease.

A FORM of chronic antimony poisoning is described by Schrumpf in the *Strassburger Med. Zeitung* as being met with in printers and typecasters. The symptomatology in such cases includes nervous troubles, somnolence, lassitude, vertigo, headache, muscular and neuralgic pains, nausea, lack of appetite, and constipation. In 120 cases examined by the author the characteristic changes in the blood and urine in poisoning by lead were wanting. Granular basophile corpuscles rarely exceeded the normal, there was a definite leucopenia and the polynuclear leucocytes were eosinophile. Red corpuscles averaged five millions, and the hæmoglobin content was from 90 to 100 per cent. There was no increase in arterial tension, and nothing abnormal could be found in the urine. Type contains about 70 per cent. of lead, 5 of zinc, and 20 of antimony, and if lead be excluded the toxic symptoms noted must be due to antimony. The author does not think that the poisonous properties of this metal are due to the arsenic with which it is usually mixed in commerce; and, moreover, two of the patients presented antimony in the fæces. As a rule it is the younger subjects who are attacked, for older men have acquired some degree of immunity. Prognosis is good generally, and subjective symptoms disappear quickly if work be stopped, a milk diet taken, and open-air exercise indulged in. As in the case of lead, personal hygiene and the washing of the hands before a meal are of the utmost importance.

### The X-rays and Sexual Sterilisation.

A SHORT account of recent work regarding the action of $x$-rays on the female internal generative organs has appeared in *La Clinique* from the pen of Dr. Perdrizet. First noticed by Schönberg, the sterilising effects of the rays were confirmed by later observers. Görl has recently published results obtained in nine cases. He has employed the treatment to overcome the nervous phenomena so often met with at the time of the menopause, and his results are worthy of notice. In two cases application of the rays brought relief, but owing to irregularity in treatment cure was incomplete. In two others, aged respectively fifty-five and fifty-six years, from fifteen to eighteen sittings sufficed to bring about cessation of the menses and the disappearance of the nervous phenomena previously noted. These cures have now lasted over a year. His fifth case is still under observation. In four other cases the treatment was instituted because of severe metrorrhagia resulting from fibro-myomata. The tumours disappeared after application of the rays. Bordier, of Lyons, has reported four cases of this last kind in which a cure resulted after a course of treatment extending over a number of weeks. The action of the rays takes place both on the ovary and on the uterine neoplasm, so that premature menopause and atrophy of the tumour are met with together. Bordier states, however, that the internal secretion of the ovary is not done away with, and consequently the treatment has an importance all its own, especially in cases where the growths are of recent formation and small size. Offergeld goes even further, and states that sterilisation of the patient is necessary in many pathological conditions, and considers that radiotherapy, which in the hands of experts is perfectly safe, is preferable to the ordinary surgical methods in vogue to secure that result, such as removal of the Fallopian tubes, etc.

### Leprosy in New Caledonia.

ACCORDING to a recent number of *Le Journal Officiel*, the progressive increase of leprosy in New Caledonia has become so marked that at last the inhabitants themselves have become alive to the necessity of undertaking preventive measures against the spread of the disease, and the Council-General of the colony has just issued a decree which, if adopted by the superior authorities, will ensure the carrying out of adequate prophylactic precautions. In December 1909 the number of lepers confined on the Ile aux Chèvres was sixty-six, of whom twelve were natives and fifty-four whites, six being either of the female sex or children. In the Bélep islands at that same date were sixty-four lepers, while nineteen were awaiting deportation at Nou island, making altogether a grand total of 137 isolated patients. The leper colonies have officially accommodation for 400 persons, of whom 219 should be housed in the Loyalty islands, but lepers are to be found anywhere and everywhere rather than in these colonies, despite the purely moral restraints imposed in these places. Unfortunately, the larger proportion of persons attacked by the disease are of European origin, and medical men practising at Nouméa and in the bush are of opinion that the disease is largely on the increase.

### Adrenalin Injections for Asthma.

THE almost magical effect which a hypodermic injection of adrenalin solution has over the paroxysms of true asthma in certain patients is by now fairly widely known. It succeeds sometimes where all other remedies fail, though, like other asthma remedies, it fails with some cases as completely and as unaccountably as it succeeds with others. The usual objection offered on hypothetical grounds to the treatment is that, should any patient become regularly addicted to it, serious danger to the heart and arteries might ensue. That this objection is not necessarily a valid or real one is shown by the history of a case of asthma published in the *Journal of the American Medical Association*. A medical man contracted whooping-cough, and during convalescence he began for the first time to suffer from bronchial asthma. All kinds of treatment were unavailing, including removal of polypi from the nose and other local treatment in that region. In little more than a year he became chronically and persistently asthmatic; slept sitting up, and seldom for more than a hour at a time. Then eight minims of adrenalin solution were given hypodermically; the symptoms vanished in half a minute. For the next five years he continued regularly to take the drug, sometimes as many as forty to sixty doses in twenty-four hours. After that, and without any change in treatment, environment, etc., he began to improve, and is now quite well; he has also regained 70 of the 84 lb. weight which he lost during the asthmatic period. The blood pressure is normal; and so are the urine, heart, lungs, retinal arteries, and every other organ. Adrenalin is now known to exert an influence on involuntary muscle fibres, and through its constricting action on the arterioles it probably does good in asthma by reducing the congestion in the bronchial mucosa.

### Excretion in the Trendelenburg Position.

Some peculiar facts have been brought to light by Dr. Bovée regarding the influence of the Trendelenburg posture on renal excretion during anæsthesia; they are published in the *American Journal of the Medical Sciences*. This observer operated on sixteen patients for whom that position was adopted, eight under ether and eight under chloroform. In each patient the normal excretion of urine *per diem* was determined at a period not too near the proposed operation. For twelve hours preceding the latter liquid food alone was allowed, and in limited quantities. The bowels were moved two or three times, the last time by an enema. No drug such as morphine, atropine, or salt solution was used before or after the operation. A catheter was passed when the administration of the anæsthetic was begun, and the bladder emptied. The catheter was then clamped, and the bladder again emptied, when the Trendelenburg position was ordered. Thereafter the urine was removed every fifteen minutes after the operation (the bladder being compressed from within the pelvis if necessary), and every fifteen minutes for two hours after the operation. The figures are quoted in detail in the original paper; they show that when in the Trendelenburg position the urine is decreased 58 per cent. under ether and 95 per cent. under chloroform. Even after striking out two patients who secreted very large quantities while receiving the anæsthetic (possibly through fright), the decrease for chloroform is still 82 per cent. The author says that this decrease cannot be due to retention of urine in the pelvis of the kidney, because for an hour and a quarter after operation the urine secreted is still less than normal, whereas it would be greatly and immediately increased if it had simply collected in the renal pelvis. He concludes that the Trendelenburg position greatly lessens the renal function, and that for those with renal inefficiency, or cardiac and arterial lesions, the Trendelenburg position is inadvisable, especially when the anæsthetic to be used is chloroform.

### Tuberculosis in New Zealand.

In a Cambridge thesis published in the *New Zealand Medical Journal*, Izard notes some interesting comparisons of tuberculosis in Europe and in that colony. First he shows that New Zealand should be but lightly scourged by tuberculosis, for the climate is very equable, and cattle can be kept in the open air all the year round; there are no slums, the population is but slightly over a million in a country as large as England and Scotland, and legislative measures against the disease have been in force for many years. All cases of tuberculosis are compulsorily notifiable and inspected by an expert who gives advice as to hygiene, ventilation, destruction of sputum, etc. Abattoirs are provided, and all meat is inspected; so also are dairies and every detected case of animal tuberculosis is followed to its source. The result of all these natural and artificial advantages is that abdominal tuberculosis is almost unknown in New Zealand; whereas in England it is still very common and has decreased by 5 per cent. in forty years, though phthisis has decreased 37 per cent. Among cattle there is but little tuberculosis, compared with its prevalence in Great Britain; but the New Zealand pigs suffer more severely. Sanitarians in the colony are not, however, satisfied with their high position of anti-tuberculous honour, but are projecting yet more stringent examinations and precautions in the endeavour to stamp out the disease.

### A Danger of Auto-serotherapy.

For some time past various clinicians have advocated the treatment of pleural effusion by injecting subcutaneously some of the fluid removed from the chest by aspiration. In the *Medical Review* a case is quoted which shows that this method is sometimes attended with serious disadvantages. A woman, aged sixty-four, was taken ill with rigors, and a pleural effusion developed. The fluid from this contained numerous lymphocytes and few polynuclears; therefore some of it was injected into a guinea-pig, as being possibly tuberculous in origin. On three occasions small quantities (about 2 c.c.) of the effusion were injected hypodermically, but without any good result; so the effusion was then aspirated away in two sittings, and it did not return. About the same date physical signs of tuberculosis developed in the right lung. The diagnosis was further confirmed by the guinea-pig developing tuberculosis and by a positive intradermic reaction. In a few weeks' time the patient developed two nodules in the spot where the pleural fluid had been injected. One was as large as a hazel nut, painless and movable; the other larger, painful, and adherent to the tissues. The former was removed entire; the latter broke down into a cold abscess. Pus from the latter caused tuberculosis in a guinea-pig, and the sinus was slow in healing. The smaller mass was found to enclose a caseating mass in the centre, and giant cells containing numerous tubercle bacilli were demonstrated. The chain of evidence seems complete, and the case is valuable as showing that autoserotherapy requires careful watching.

